# Assessment of Immunohistochemical Expression of Cluster of Differentiation 44 (CD44) in Breast Carcinoma

**DOI:** 10.7759/cureus.91104

**Published:** 2025-08-27

**Authors:** Ranu Kumari, Satish G Arakeri, Sai Kulkarni

**Affiliations:** 1 Pathology, Shri B. M. Patil Medical College, Hospital and Research Centre, Bijapur Lingayat District Educational (BLDE) Association (Deemed to be University), Vijayapura, IND

**Keywords:** carcinoma of breast, cd44+, estrogen receptor (er), lymph node metastasis, progesterone receptor (pr)

## Abstract

Cluster of differentiation 44 (CD44) expression in breast carcinoma is associated with basal-like epithelial markers in tumor cells, advanced tumor stage, and metaplastic variants. Since CD44 is a recently identified marker, further research is necessary to understand its immunoexpression and its relationship with hormone receptor status and other prognostic factors that influence patient management and therapy. This study was conducted to assess the expression of CD44 in breast cancer tissue.

A cross-sectional investigation was conducted in our hospital on 60 mastectomy specimens received in the histopathology section of the Department of Pathology. The patient's age, tumor size, histological type, histological grade, and lymph node status were recorded. Immunohistochemical staining for estrogen receptor (ER), progesterone receptor (PR ), human epidermal growth factor receptor 2/neu (HER2/neu), and CD44 markers was performed, and expression of CD44 was correlated with these clinicopathological and prognostic parameters. The results were subjected to statistical analysis.

CD44 expression was detected in 51 of the 60 cases (85%). A statistically significant correlation was found between the expression of CD44 with patients’ age, size of tumor, histological grade, and HER2/neu hormonal marker. No statistically significant relationship was observed between other factors, such as lymph node status and the expression of ER and PR.

The expression of CD44 showed a strong correlation with known poor prognostic indicators, such as HER2/neu status and histological grade. Therefore, CD44 expression indicates an aggressive tumor biology and can serve as an independent marker for prognosis and therapy.

## Introduction

Breast cancer is the primary cause of cancer-related mortality for women and one of the most often diagnosed cancers [1]. Every year, over 1 million fresh cases of breast cancer are identified, making up more than 23% of all cancers that affect women worldwide [1]. Less developed regions of Southern and Eastern Asia, as well as sub-Saharan Africa, have lower rates of breast cancer [2]. Breast cancer is the most frequently diagnosed cancer worldwide, with an estimated 2.6 million new cases, representing approximately 11.6% of all cancer diagnoses, according to GLOBOCAN 2022 estimates [3]. As per the World Health Organization (WHO) 2022, there are significant variations in breast cancer rates based on human development worldwide.

The cause and pathophysiology of breast cancer interact, making it a complex illness influenced by several genetic, hormonal, and environmental factors [4]. Etiological factors include a positive family history, consumption of alcohol, early menarche, late menopause, a sedentary lifestyle, nulliparity, and hormone replacement therapy [5]. Numerous variables, including tumor size, histologic type, histologic grade, age at diagnosis, pTNM (pathological Tumor, Node, Metastasis) staging, lymph node metastasis, and molecular profiles (such as the p53 gene, human epidermal growth factor receptor 2/neu (HER2/neu), and others), influence the prediction of breast cancer [1,6]. The standard immunohistochemical markers are estrogen receptor (ER), progesterone receptor (PR), and HER2/neu (human epidermal growth factor receptor 2/neu). Breast cancer is divided into four main groups depending on gene expression profiling studies: HER2 type, basal-like/triple negative, luminal A, and luminal B [6].

Cancer stem cells (CSCs) are presently observed as the precursors for any malignancy. There are several CSCs identified in solid epithelial malignancies, such as breast cancer. CD24, CD44, and aldehyde dehydrogenase are a few examples. Breast CSCs are typically characterized by a CD44⁺/CD24⁻/low phenotype, which is associated with increased tumor-initiating potential and self-renewal capacity [7]. CD44 is a transmembrane glycoprotein that crosses the cell membrane and binds to hyaluronic acid, resulting in the activation of various cascades. Its expression maintains the stemness of tumor cells as a good promotion of tumorigenesis [8].

There are 20 exons in the CD44 gene. Its N-terminal and C-terminal domains are encoded by constant exons 1-5 and 16-20, respectively. The most prevalent type of CD44 has ten constant exons [9]. CD44 in tumor cells of breast cancer, when compared with luminal A, B, HER2/neu+, and basal-like classification, it was observed that the basal-like variant showed more expression of CD44 when compared to almost Nil expression of CD44 in luminal A, as well as luminal B. Hence, CD44 expression suggests poor prognosis [10].

## Materials and methods

This cross-sectional study was conducted on 60 patients who were diagnosed with invasive breast carcinoma of no special type (NST). The study period was from May 1, 2023, to December 31, 2024. It was carried out in the Histopathological Section, Department of Pathology, Shri B. M. Patil Medical College, Hospital and Research Centre, Bijapur Lingayat District Educational (BLDE) Association (Deemed to be University), Vijayapura, Karnataka, India. Institutional Ethical Committee approval was obtained from BLDE (Deemed to be University) on April 10, 2023, under the approval letter number BLDE (DU)/IEC/926/2023-24. All modified radical mastectomy specimens of breast cancer received in the histopathology section were studied. Exclusion criteria included breast biopsy, unfixed, and lumpectomy specimens.

The tissue was processed in 10% neutral buffered formalin. Sections having a thickness of four microns were cut from each tissue block. One section was stained with hematoxylin and eosin (H&E) for histopathological diagnosis. Four more sections from the same block were placed on a poly-L-lysine-coated slide from paraffin-embedded tissue blocks. These sections were then used for immunohistochemical staining of the CD44, HER2/neu, progesterone receptor (PR), and estrogen receptor (ER). The primary antibodies used were monoclonal antibody (Clone DF1485, ready-to-use) for CD44, monoclonal antibody (Clone EP1, ready-to-use) for ER, monoclonal antibody (Clone 1E2, ready-to-use) for PR , and monoclonal antibody (Clone 4B5, ready-to-use) for HER2/neu. The expression of ER and PR was assessed using the Allred scoring system, which combines a proportion score (ranging from 0 to 5) and an intensity score (ranging from 0 to 3). A total score of less than 2 was considered negative, and a score of 2 or more was considered positive. The maximum possible score was 8. HER2/neu expression was interpreted according to ASCO guidelines. A score of 0 indicated no or faint membrane staining in ≤10% of tumor cells, and 1+ indicated faint/incomplete membrane staining in >10% of tumor cells- both considered negative. A score of 2+ represented moderate, complete membrane staining in >10% of tumor cells (equivocal), and 3+ indicated complete, intense circumferential staining in >10% of tumor cells (positive).

The expression of CD44 by immunohistochemistry was linked to predictive factors such as tumor size, lymph node status, histological grade, as well as ER, PR, and HER2/neu status. Histological grading was performed using the Modified Bloom-Richardson grading system. This system assesses three morphological criteria: tubule formation, nuclear pleomorphism, and mitotic count. Each is scored from 1 to 3, and the total score determines the tumor grade: Grade I (3-5), Grade II (6-7), and Grade III (8-9).

The staining intensity and pattern of CD44 expression were examined in malignant tumor cells. CD44 IHC with either partial or complete membrane staining was considered positive, whereas CD44 was considered negative when it showed negative or no membrane staining. The staining intensity was semi-quantitatively graded as weak, moderate, or strong based on visual assessment. CD44 was considered negative when there was no detectable membranous staining.

Data were entered into a Microsoft Excel sheet (Microsoft Corp., Redmond, WA) and analyzed using SPSS version 20 (IBM Corp., Armonk, NY). Normally distributed continuous variables between the two groups were compared using an independent sample t-test. For variables that do not follow a normal distribution, the Mann-Whitney U test was utilized. Categorical variables between the two groups were compared using the chi-square test and Fisher's exact test. *P* <0.05 was considered statistically significant. Two-tailed statistical tests were performed.

## Results

**Figure 1:**
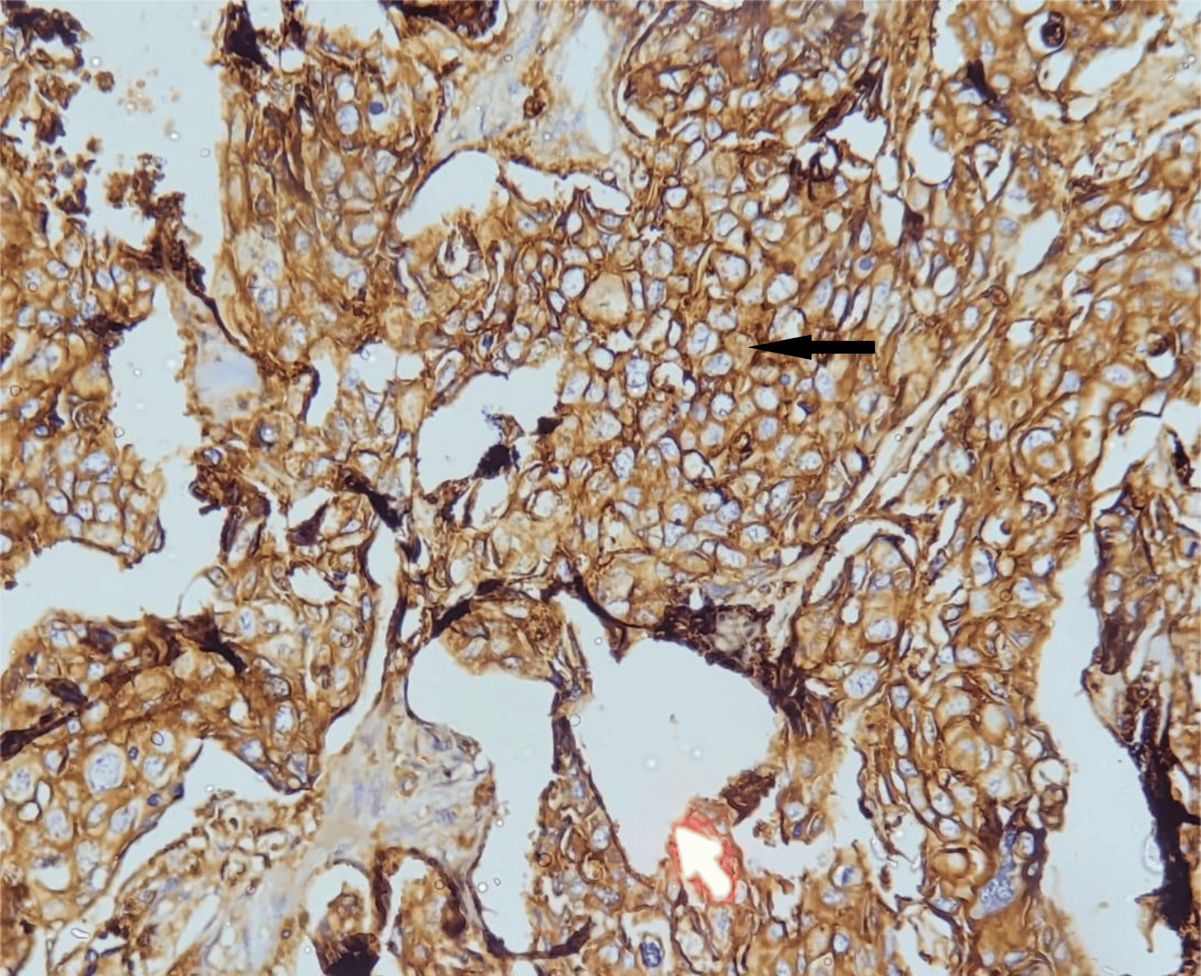
Microphotograph of immunohistochemical marker CD44 showing complete cytoplasmic staining in invasive breast carcinoma NOS (200x). CD, cluster of differentiation; NOS, not otherwise specified

**Figure 2:**
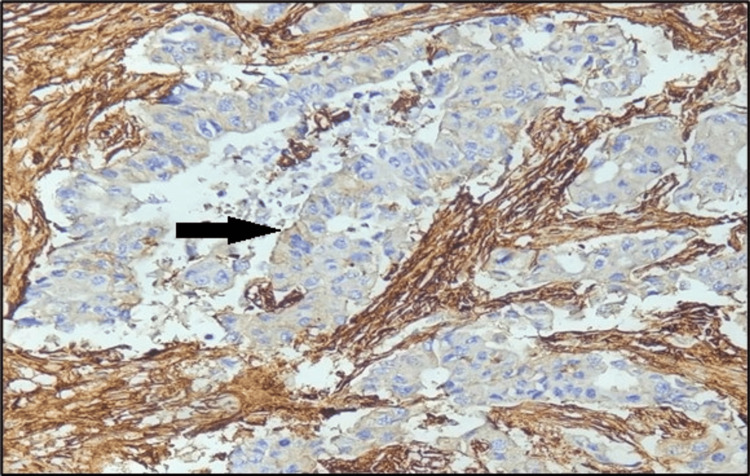
Microphotograph of immunohistochemical marker CD44 showing focal cytoplasmic staining in invasive breast carcinoma NOS (200x). CD, cluster of differentiation; NOS, not otherwise specified

The current study involved 60 patients with an invasive breast cancer diagnosis. In malignant tumor cells, the staining pattern and intensity of CD44 were assessed. All CD44-positive cases showed membranous positivity. CD44 immunohistochemistry showing either complete membranous staining (Figure 1) or focal membranous staining (Figure 2) was considered positive. CD44 positivity was seen in 51 (85%) cases, and the remaining 9 (15%) cases were CD44 negative, which showed no membrane staining. 

The age of patients with invasive breast carcinoma ranged from 30 to 80 years, with a mean age of 54.5 years and a median age of 55 years.

**Figure 3:**
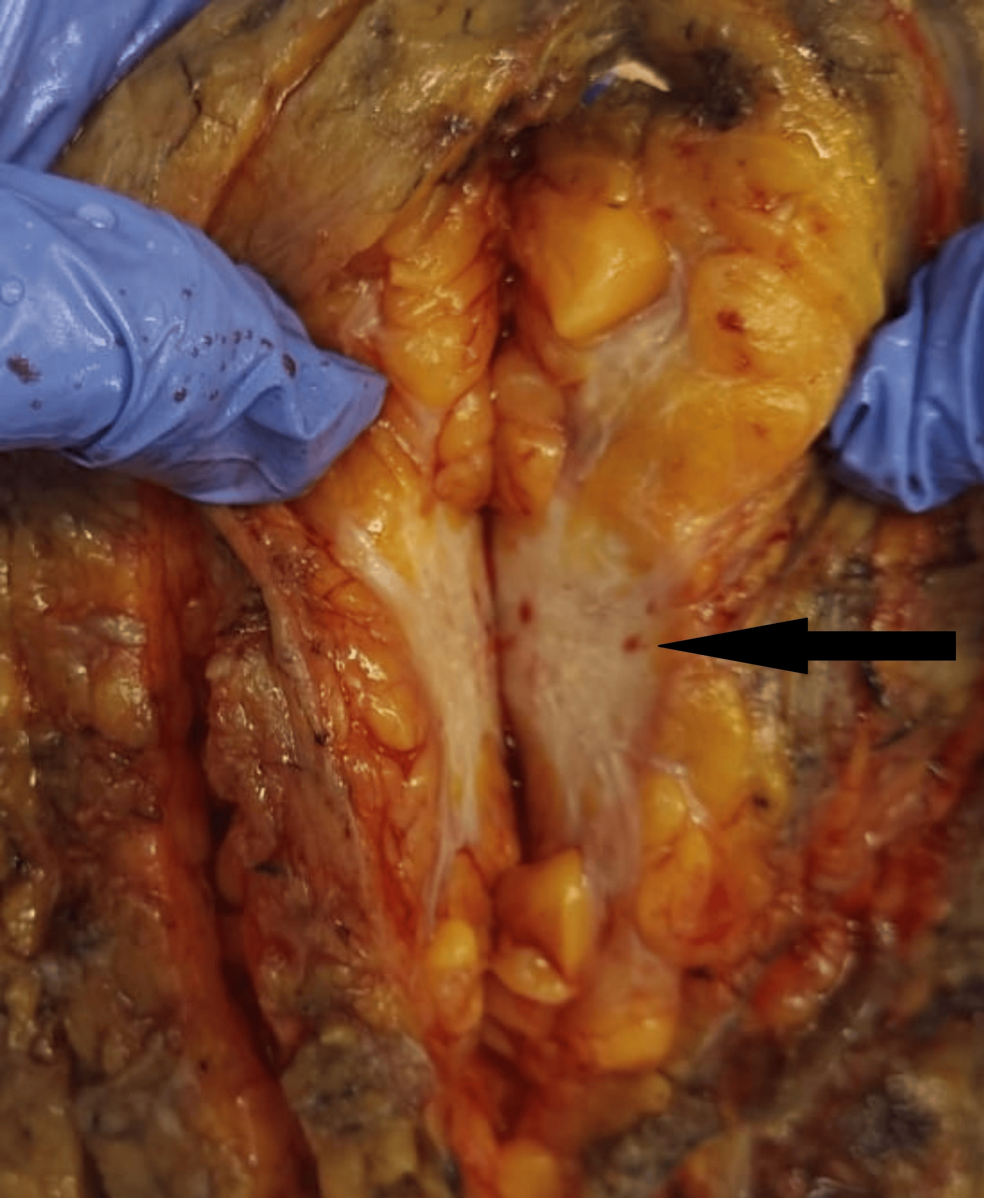
Macrophotograph showing a cut section of invasive breast carcinoma with a tumor size of 3 × 2.5 × 1 cm.

The size of the tumor ranged from 1 to 10 cm. Twenty-four (47%) cases with CD44 positivity were in the group of tumor size T2 (Figure 3), followed by 14 (27.4%) cases in T1 and 8 (15.6%) cases in T3. The fewest CD44-positive cases were in T4, with 5 (9.8%) cases. Among CD44-negative cases, 4 (44%) were in T2, followed by 2 (22.2%) each in T1 and T4, and 1 (11.1%) in T3.

The *P*-value was 0.002, indicating a highly statistically significant association between CD44 expression and tumor size (Table 1).

**Table 1:** Correlation of CD44 expression with tumor size. The *P*-value of 0.002 was considered significant. *Statistically significant. CD44, cluster of differentiation 44

Parameters	CD44		Student’s t-test	Mann-Whitney U test	*P*-value
	Negative, *n* (%)	Positive, *n* (%)			
Tumor size					
T1	2 (22.2%)	14 (27.4%)	-3.20	180	0.002*
T2	4 (44%)	24 (47%)			
T3	1 (11%)	9 (15%)			
T4	2 (22%)	5 (9.8%)			

**Figure 4:**
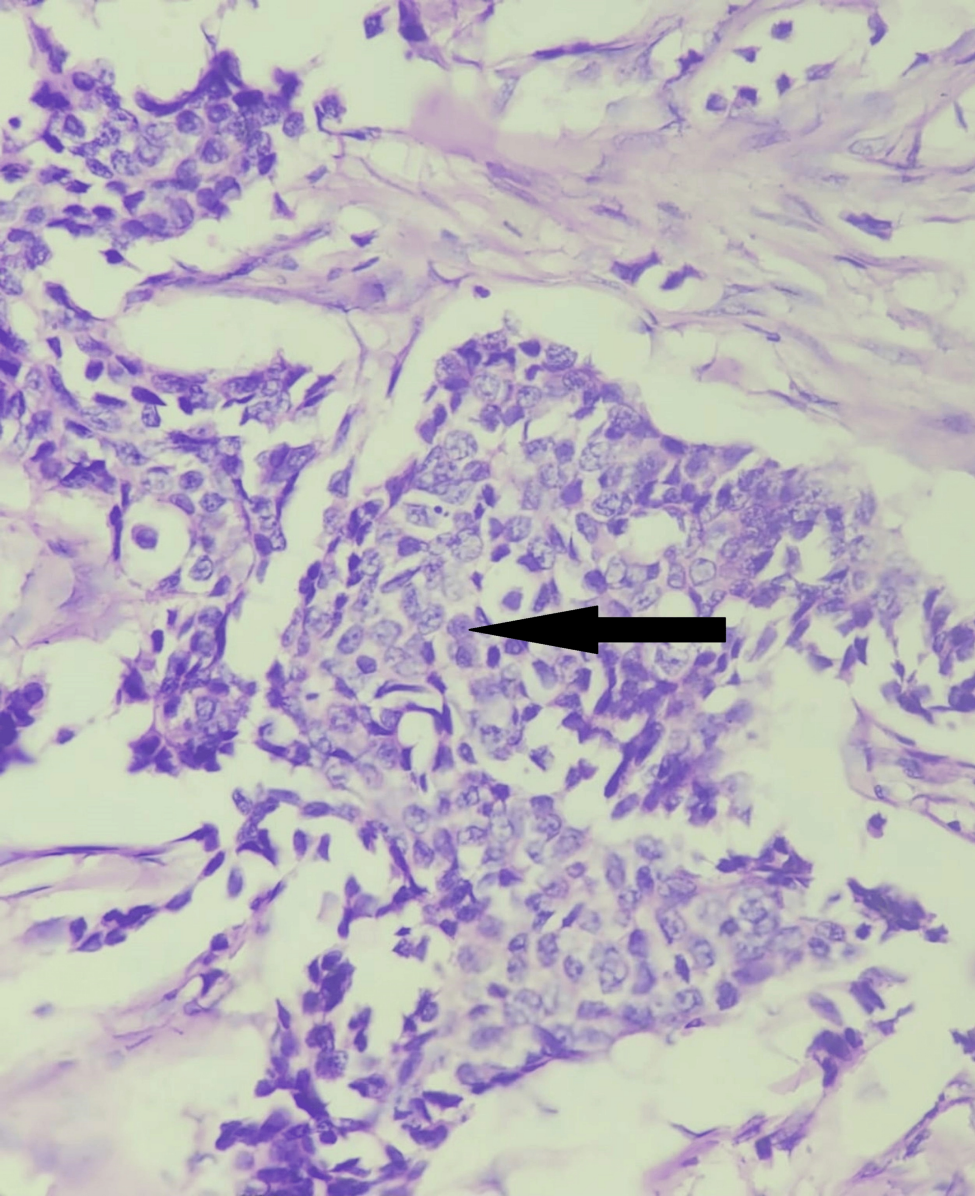
Microphotograph showing infiltrating tumor cells arranged in sheets with moderate nuclear pleomorphism (H&E, 200×). H&E, hematoxylin and eosin

The majority of cases in this study with positive CD44 expression were associated with Grade 2 (Figure 4), i.e., 26 (51%) cases, followed by Grade 1 with 16 (31.3%) cases and Grade 3 with 9 (17%) cases. Among CD44-negative cases, 6 (66.6%) were in Grade 2, followed by 2 (22.2%) in Grade 1 and 1 (11.1%) in Grade 3. The *P*-value was 0.02, indicating a statistically significant correlation between CD44 expression and the histological grade of the tumor (Table 2).

**Table 2:** Correlation of CD44 expression with histological grading. The *P*-value of 0.022 was considered significant. *Statistically significant. CD44, cluster of differentiation 44

Histological grade	CD44			Student’s t-test	Mann-Whitney U test	*P*-value
	Negative	Positive	Total			
Grade 1	2 (22.1%)	16 (31.3%)	18 (30%)	-2.27	248	0.022*
Grade 2	6 (66.6%)	26 (51%)	32 (53.3%)			
Grade 3	1 (11.1%)	9 (17%)	10 (16.6%)			
Total	9 (100%)	51 (100%)	60 (100%)			

In this study, among 60 cases of invasive breast carcinoma, 40 (66.6%) had definite nodal status. Of these, 33 (82.5%) were CD44-positive and 7 (17.5%) were CD44-negative. Twenty cases (33%) lacked lymph node metastases; 18(35%) cases showed CD44 expression. The *P*-value was 0.44, indicating no statistically significant correlation between CD44 expression and lymph node status.

Of 22 ER-negative cases, 17 (33.3%) presented with CD44 positivity. Among 38 cases with positive ER expression, 34 (66.6%) cases showed CD44 expression. The *P*-value was 0.2, indicating no statistically significant correlation between CD44 expression and ER status of the tumor.

Of 24 PR negative cases, 19 (37.2%) cases showed CD44 positivity. Of 36 cases with positive PR expression, 32 (62.6%) cases showed CD44 positivity. The *P*-value was 0.30, indicating no statistically significant correlation between CD44 expression and PR status of the tumor.

**Figure 5:**
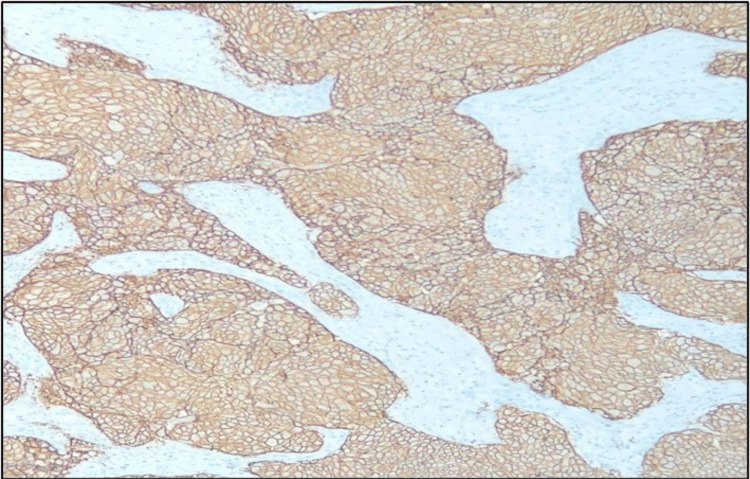
Microphotograph showing membranous positivity for HER2/neu in invasive breast carcinoma, not otherwise specified (NOS) (200×). HER2/neu: human epidermal growth factor receptor 2/neu

Of the 30 cases with HER2/neu-negative status (Figure 5), 28 (55%) showed CD44 positivity. Among the 28 cases with HER2/neu-positive expression, 21 (41%) were CD44-positive. The *P*-value was 0.04, indicating a statistically significant correlation between CD44 expression and HER2/neu status of the tumor (Table 3).

**Table 3:** Correlation of CD44 with HER2/neu receptor status. The *P*-value of 0.04 was considered significant. *Statistically significant, degrees of freedom (df): 2, effect size (Cramer's V) ≈ 0.26. CD44, cluster of differentiation 44; HER2/neu, human epidermal growth factor receptor 2/neu

HER2/neu status	CD44			Chi-square test	*P*-value
	Negative, *n* (%)	Positive, *n* (%)	Total, *n* (%)		
Equivocal	0 (0.0%)	2 (4%)	2 (3.3%)	4.18	0.04*
Negative	2 (22.2%)	28 (55%)	30 (50%)		
Positive	7 (78%)	21 (41%)	28 (47%)		
Total	9 (100%)	51 (100%)	60 (100%)		

Comparison of age, histological type, lymph node status, ER, PR, and HER2/neu with CD44 is shown in Table 4. 

**Table 4:** Comparison of CD44 with age, histological type, lymph node status, ER, PR, and HER2/neu status. *P*-values of 0.008 and 0.04 were considered significant. CD44, cluster of differentiation; IDC-NOS, infiltrating ductal carcinoma-not otherwise specified; ILC, invasive lobular carcinoma; IPC, invasive papillary carcinoma; EPC, encapsulated papillary carcinoma; MUC C, mucinous carcinoma; ER, estrogen receptor; PR, progesterone receptor; HER2/neu, human epidermal growth factor receptor 2/neu

Parameters	CD44		Chi-square test	*P*-value	Degrees of freedom	Effect size
	Negative, *n* (%)	Positive, *n* (%)				
Age (years)						
<30	0 (0%)	0 (0%)	6.99	0.008	4	0.341
31-40	2 (22.2%)	6 (12%)				
41-50	4 (4.44%)	8 (15.6%)				
51-60	0 (0%)	18 (35%)				
>60	3 (33%)	19 (37.2%)				
Histological types						
IDC-NOS	9 (100%)	45 (88.2%)	1.17	0.27	4	0.139
ILC	0 (0.0%)	2 (3.3%)				
EPC	0 (0.0%)	2 (3.3%)				
IPC	0 (0.0%)	1 (2%)				
MUC C	0 (0.0%)	1 (2%)				
Lymph node status						
Involved	7 (77.7%)	33 (65%)	0.58	0.44	1	0.098
Not involved	2 (22.2%)	18 (35%)				
ER status						
Negative	5 (55.5%)	17 (33.3%)	1.62	0.20	1	0.164
Positive	4 (44.4%)	34 (66.6%)				
PR status						
Negative	5 (55.5%)	19 (37.2%)	1.06	0.30	1	0.133
Positive	4 (44.4%)	32 (62.6%)				
Her2/neu						
Equivocal	0 (0.0%)	2 (4%)	4.18	0.04	2	0.264
Negative	2 (22.2%)	28 (55%)				
Positive	7 (78%)	21 (41%)				

Comparison of histological grade and tumor size with CD44 is depicted in Table 5.

**Table 5:** Comparison of CD44 with histological grade and tumor size. Both parameters show a statistically significant correlation (*P* = 0.02 and *P* = 0.002; *P* < 0.05 considered significant). CD44, cluster of differentiation 44

Parameters	CD44		Student’s t-test	Mann-Whitney U test	*P*-value
	Negative, *n* (%)	Positive, *n* (%)			
Histological grade					
I	2 (22.1%)	16 (31.3%)	-2.27	248	0.022
II	6 (66.6%)	26 (51%)			
III	1 (11%)	9 (17%)			
Tumor size					
T1	2 (22.2%)	14 (27.4%)	-3.20	180	0.002
T2	4 (44%)	24 (47%)			
T3	1 (11%)	9 (15%)			
T4	2 (22%)	5 (9.8%)			

## Discussion

Breast cancer is the most widespread form of carcinoma among women in both high- and low-resource environments, accounting for 2.6 million new cases, representing approximately 11.6% of all cancer diagnoses globally [3,11]. A comprehensive search for prospective disease markers is necessary to develop tailored care, especially for those with therapeutic and prognostic implications [12]. To ascertain whether CD44 is associated with a particular clinicopathological feature and hormonal state, as well as whether it is an independent prognostic marker, this study was carried out to evaluate CD44 expression in instances of breast cancer.

This study included a total of 60 cases of invasive breast carcinoma, with patient ages ranging from 30 to 80 years. The mean age was 54.5 years, and the median age was 55 years.

The highest number of CD44-positive cases was observed in patients older than 60 years, with 19 of 22 cases (37.2%), followed by 18 cases (35.2%) in the 51-60 year age group. The *P*-value was 0.008, indicating a statistically significant correlation between CD44 expression and patient age. Similar findings were reported by Elbaiomy et al. [7] and Honeth et al. [13], who observed the highest number of cases in the 50-70 year age group (43 cases, 56.6%), followed by the 30- to 50-year age group (33 cases, 43.4%), with a median age of 50 years.

The following histological types were included according to the WHO classification: invasive breast carcinoma of no special type (IBC-NST), invasive lobular carcinoma (ILC), encapsulated papillary carcinoma (EPC), and invasive papillary carcinoma (IPC). The majority of cases were IBC-NST, i.e., 54 out of 60 cases (90%). However, no histological types showed statistically significant differences in CD44 expression. In a study by Honeth et al. [13], the majority of cases were invasive breast carcinoma (176 out of 240), showing a statistically significant correlation (*P* < 0.05). 

In this study, among 40 cases with nodal metastasis, 33 (65%) exhibited CD44 positivity. However, the analysis showed no statistically significant correlation between CD44 expression and lymph node metastasis (*P* = 0.44). In studies by Honeth et al. [13] and Roosta et al. [14], similar findings were reported. Honeth et al. observed CD44 positivity in approximately 60% of breast cancer cases, particularly in the basal-like subtype [13]. Roosta et al. reported that 45 of 73 cases of lymph node metastasis (61%) showed CD44 positivity [14]. However, no statistical correlation was found in these two studies. The existence of nodal metastasis is linked to the likelihood of distant metastasis. Recurrence after 10 to 25 years post-diagnosis has been associated with a higher initial involvement of lymph nodes, with rates varying from 12.7% to 24.6% [15].

Tumor sizes in this study ranged from 1 to 10 cm. In this study, the majority of cases, i.e., 28 (46.6%), had tumor sizes between 2 and 5 cm (T2) and showed CD44 positivity, with a statistically significant correlation (*P* < 0.05). In contrast, in studies conducted by Roosta et al. and Zou et al. [14,16], the highest number of cases also belonged to size T2 (2-5 cm), with 59 out of 100 cases and 32 out of 51 cases, respectively, but the findings were not statistically significant (*P* > 0.05).

In the studies conducted by Elbaiomy et al. and Korfias et al. [7,17], CD44 expression in the positive group was found to be associated with a higher tumor grade (Grade 3), with a *P*-value of 0.001. In this study, Grade 2 tumors constituted the majority (53.3%) and showed a statistically significant correlation (*P* < 0.05). The same findings were observed in studies conducted by Roosta et al. and Tomar et al. [14,18], where 82 of 100 cases (82%) and 40 of 58 cases (69%), respectively, were of histological Grade 2. Neither study found a statistically significant correlation between CD44 and histological grade, which is consistent with their observations.

According to this study, there is a highly significant correlation between CD44 with HER2/neu expression and an insignificant association with positive ER and positive PR expression. In the current study, CD44 was statistically significant with HER2/neu expression, showing a *P*-value of 0.04. A similar finding was seen in a study done by Honeth et al. [13], where 38 out of 240 (16%) cases showed HER2/neu positivity (*P*-value 0.002). Our results contradicted those of studies conducted by Elbaiomy et al. and Voutilainen et al. [7,19], where 7 of 32 cases (22%) and 2 of 62 cases (3%), respectively, showed HER2/neu positivity, but the findings were not statistically significant, with *P*-values of 0.006 and 0.417.

Limitations of this study

Due to diversity in the expression of CD44 with different parameters, a study with a greater sample size is needed. A deeper comprehension of CD44 expression and its relationship to clinicopathological characteristics will be possible with more research, including bigger sample sizes.

Recommendations

Additional research is required to ascertain the precise prognostic significance of the CD44 marker, an effective therapeutic approach that aims to eliminate cancer stem cells by targeting these cells, as it has an increased risk of tumor recurrence.

## Conclusions

In this study, CD44 expression was associated with poor prognostic factors in breast cancer, including histological grade, tumor size, and HER2/neu status. Most CD44-positive cases had tumors measuring 2-5 cm, were classified as Grade 2, and predominantly showed negative HER2/neu expression. This suggests that CD44 expression is linked to a bad prognosis. Nonetheless, no meaningful statistical relationship was identified among other clinicopathological and prognostic factors such as histological type, lymph node metastasis, and ER/PR status. Given that CD44 expression supports the stemness of tumor cells and significantly enhances tumorigenesis, it can be considered a valuable prognostic and therapeutic marker for guiding future treatment strategies.
